# Circulating miRNA-192 and miR-29a as Disease Progression Biomarkers in Hepatitis C Patients with a Prevalence of HCV Genotype 3

**DOI:** 10.3390/genes14051056

**Published:** 2023-05-08

**Authors:** Amin Ullah, Irshad Ur Rehman, Katharina Ommer, Nadeem Ahmed, Margarete Odenthal, Xiaojie Yu, Jamshaid Ahmad, Tariq Nadeem, Qurban Ali, Bashir Ahmad

**Affiliations:** 1 Department of Health and Biological Sciences, Abasyn University, Peshawar 25000, Pakistan; 2 Institute for Pathology, University of Cologne, 50923 Cologne, Germany; 3 Center of Biotechnology and Microbiology, University of Peshawar, Peshawar 25000, Pakistanbashirdr2015@yahoo.com (B.A.); 4 Institute of Transfusion Medicine, University of Cologne, 50923 Cologne, Germany; 5 Center of Excellence in Molecular Biology, University of the Punjab, Lahore 54000, Pakistan; 6 Department of Plant Breeding and Genetics, University of the Punjab, Lahore 54000, Pakistan

**Keywords:** hepatitis C virus, liver injury, microRNA-192, microRNA-29a, HCV genotype-3

## Abstract

MicroRNAs miR-29a and miR-192 are involved in inflammatory and fibrotic processes of chronic liver disease, and circulating miR-29a is suggested to diagnose fibrosis progression due to hepatitis C virus (HCV) infection. This study aimed to evaluate the expression profile of circulating miR-192 and 29a in a patient cohort with a high frequency of HCV genotype-3. A total of 222 HCV blood samples were collected and serum were separated. Patients were classified into mild, moderate, and severe liver injury based on their Child–Turcotte–Pugh CTP score. RNA was isolated from the serum and used for quantitative real-time PCR. The HCV genotype-3 (62%) was the predominant HCV genotype. In HCV patients, the serum miR-192 and miR-29a levels were significantly upregulated in comparison to healthy controls (*p* = 0.0017 and *p* = 0.0001, respectively). The progression rate of miR-192 and 29a in the patient group with mild was highly upregulated compared to patients with moderate and severe hepatitis infection. The ROC curve of miR-192 and miR-29a of moderate liver disease had a significant diagnostic performance compared to the other HCV-infected groups. The increase in miR-29a and miR-192 serum levels was even slightly higher in patients with HCV genotype-3 than in non-genotype-3 patients. In conclusion, serum miR-192 and miR-29a levels significantly increased during the progression of chronic HCV infection. The marked upregulation in patients with HCV genotype-3 suggests them as potential biomarkers for hepatic disease, independently of the HCV genotype.

## 1. Introduction

Worldwide, the hepatitis C virus (HCV) is a principal causative agent of human liver infection. It is estimated that 180 million people are infected with HCV globally each year. Chronic HCV infection causes liver failure and hepatocellular carcinoma (HCC), and in North America, it is the primary indication for liver transplantation at present [1]. HCV consists of 9600 nucleotides, with a single-stranded RNA genome containing a single large open reading frame (ORF) [2,3]. Genetic variations are classified into six main genotypes, which include several subtypes. Genotypes 1, 2, and 3 are the most common genotypes, accounting for more than 80% of HCV infections worldwide. In the case of North America and Europe, genotype-1 is predominant, and in Southeast Asia, genotype-3 is the most prevalent, including subtypes 3a, 3b, 3c, 3d, 3e, and 3f [4,5]. HCV infections result in chronic liver inflammatory changes, including liver fibrosis and cirrhosis [3]. Metabolic changes often accompany liver fibrosis, and 70% of HCV infections lead to steatosis. Interestingly, hepatic fat accumulation occurs more frequently and is much more pronounced in patients infected with genotype-3 [6,7].

Most screening studies for the non-invasive marker, indicating the progression of hepatitis-C-caused chronic inflammation, were performed on cohorts with a prevalence of genotype-1, and differences depending on the genotypes are not known. Circulating microRNAs (miRNAs) released from the injured liver are regarded as suggestive indicators of inflammation-mediated disease severity and fibrosis [8,9].

MicroRNAs are small, non-coding moieties of approximately 19–22 nucleotide-long RNA molecules. They are involved in gene expression, targeting mRNA and attenuating translation. MicroRNAs are found in organisms ranging from unicellular to high-multicellular organisms, including mammals and plants. In the case of mammals, miRNAs regulate 60% of the protein-coding genes [10,11,12]. During maturation, mature miRNAs are generated by successive cleavages of Pol-II-transcribed primary RNA precursors by Drosha and Dicer [10,11,12]. The RNA-induced silencing complex (RISC) enables microRNAs to modulate gene expression via translational repression and mRNA degradation, with the latter mechanism mediating target inhibition more effectively [2]. Due to their ability to target most mRNAs in the human genome, microRNAs play crucial roles in biological processes, including development, immunity, cancer, and pathogen infections. In addition, miRNA expression is highly tissue-specific, and various human diseases have been linked to altered miRNA expression patterns [13].

Their dysregulation is linked with human pathologies, including cardiovascular diseases, neurological disorders, and chronic liver disease and development of hepatocellular carcinoma (HCC) [8,9,14].

Importantly miRNAs are released from cells by active secretion or passive release uponcell damage [15]. The potential application of extracellular miRNAs released into the bloodstream as non-invasive biomarkers is intensively studied. Various studies have examined the efficacy of microRNAs in diagnosing HCC. The miR-155 and miR-146a have been identified as dysregulated in patients with HCV-associated hepatocellular carcinoma [16,17,18,19]. Additionally, a combination of four miRNAs (miR-21, miR-122, miR-192, and miR-223) was highly accurate in diagnosing chronic hepatitis [19,20]. MicroRNAs have also been investigated as a potential diagnostic tool for HCV infection, despite the limitations of current diagnostic methods, which include a high rate of false negatives during the early stages of infection [21]. Thus, some studies have demonstrated that patterns of microRNA expression can distinguish between individuals with chronic HCV infection and healthy controls, as well as between patients with varying stages of liver disease. MiR-122, a liver-specific miRNA, regulates HCV replication, and its downregulation increases viral load and disease progression [19]. MiR-205, downregulated in most liver diseases, could be a diagnostic and treatment molecule. MiR-29a, miR-146a, and miR-155 also modulate HCV replication and host immune response [22]. The microRNA-29a (miR-29a) is a member of miR-29 family, whose serum levels are increased upon HCV mediated liver injury [23]. Importantly, miR-29 targets transcripts encoding ECM proteins, such as fibrillins, β1 integrin, laminins, collagens, and elastin [24,25,26]. Studies on western patient cohorts with a high prevalence of HCV genotype-1 infections also showed a change in circulating miR-29a serum levels, whereas hepatic tissue levels were reduced [23,26].

Another interesting fact is the close link between miR-192 expression and TGF-β1 signaling during renal fibrosis [27]. Furthermore, miR-192 is a hepatic-enriched miRNA recognized to be upregulated in the serum of HCV patients. Thus, miR-192 is suggested to be involved in processes mediating HCV-infection-associated liver disease and to serve as a potential target against viral pathogenesis [28]. Interestingly, miR-192, in combination with other miRNAs, is used as a novel biomarker for steatosis associated liver injury [28,29].

MicroRNA-192 and miR-29a have been proposed as biomarkers of liver injury in patients with chronic HCV infection. As previous reports have included patients infected with HCV genotype 3 to a limited extent, we focused our study on the circulating profiles of miR-192 and miR-29a in HCV patients with a high prevalence of HCV genotype 3 infection.

## 2. Materials and Methods

### 2.1. Sample Collection

The patient’s samples and data, containing demographic, clinical characteristics and estimated infection time, were collected by Hayatabad Medical Complex (HMC) and Khyber Teaching Hospital (KTH) Peshawar, Pakistan. The demographic characteristics are shown in Supplementary Table S1A–C.

The HCV genotyping was performed as previously designated by Ullah et al. [2]. The Ethical Committee approved the study, Center of Biotechnology & Microbiology (COBAM), University of Peshawar, Pakistan, and written informed consent was obtained from all the recruited patients. That the experimental samples, including the collection samples material, were confirmed to comply with relevant institutional, national, and international guidelines and legislation with appropriate permissions from authorities of the Department of Health and Biological Sciences, Abasyn University, Peshawar, Pakistan and Center of Biotechnology and Microbiology, University of Peshawar, Peshawar, Pakistan for the collection of sample specimens.

We included patients who showed PCR positivity for the 5′ untranslated region (UTR) of the HCV RNA genome. Patients who were co-infected with other virus types such as HAV or HBV or HDV were excluded. Furthermore, patients were excluded when their viral load was below a titre 500 IU/mL and if the RNA quality in serum was shown to be insufficient. 

### 2.2. RNA Isolation

HCV patients’ blood samples were centrifuged, and the serum was taken. RNA was extracted from 200 µL serum using the Qiagen Kit (Qiagen, Hilden, Germany. Cat No./ID: 217204) according to the manufacturer’s protocol and as described earlier. Before the RNA extraction, SV40-miRNA (5-UGAGGGCUGAA AUGAGCCUU-3) (Qiagen, Hilden, Germany, Cat No./ID: 331535) was spiked in (2 fmol/200 µL serum) for the later normalization of the miRNA-192 and miR-29a levels [19,30,31].

### 2.3. Synthesis of cDNA and RT-PCR

The complementary DNA (cDNA)was prepared using the miScript-Reverse Transcription Kit (Qiagen kit. Cat No./ID: 218160) as described earlier For real-time PCR, the miRNA-SYBR Green PCR Kit and SV-40 primers, miRNA-192, and miR-29a from Qiagen (Hilden, Germany) were used. Thermo-cycling conditions of the RT-PCR were as follows: initial denaturation 95 °C/3 min, following cycles of PCR template denaturation 94 °C/30 s, annealing 55 °C/45 s and extension 70 °C/45 s. All the steps were performed in triplicate and in agreement with the supplier’s guidelines. A dilution series was generated, and miRNA levels were quantified using the standard curves. Spike-in SV40-miRNA quantification was used to normalize miR-29a and miR-192 extracellular miRNAs levels [19,31,32].

### 2.4. Data Analysis

The data analysis was conducted using GraphPad Prism 5 and IBM SPSS software 25.0. One-way ANOVA test and *t*-test were used for the statistical significance, indicated as *p*-value. The non-parametric counterpart of the ANOVA, which was obtained using Kruskall–Wallis test, was also employed in cases of deviation from the normality assumption. Bartlett’s test was used for equal variance and a strong significant correlation between variables. ROC and AUC analyses were conducted to determine diagnostic performance and the significance of microRNA expression. The best cut-offs were selected by maximizing the Youden index; that is, the sum of specificity and maximized sensitivity. Pearson’s correlation was performed between the two variables. Values of less than 0.05 were considered statistically significant (p).

## 3. Results

### 3.1. Characteristics of the Patient Cohort

The HCV patients (n = 222) were grouped into mild, moderate, and severe based on the Child–Turcotte–Pugh (CTP) score. Liver disease progression was classified into mild, moderate, and severe, and the score system was as follows: 5–6 was referred to as Child Class A (mild), 7–9 as Child Class B (moderate) and 10–15 as Child Class C (severe) [9]. The patients were admitted to Peshawar’s tertiary hospitals from April 2016 to October 2018. The patients’ medical data and demographic summary are presented in Supplementary Table S1A,B. The mean age of the patients was 49.03 ± 12.65 years. Importantly, there were significant differences (*p* < 0.0001) in the alanine transaminase enzyme (ALT) and α-fetoprotein (AFP) levels of patients with progressive chronic HCV liver disease. The controls (n = 52) were healthy blood donors, and they are described in Supplementary Table S1C. Notably, in our study, HCV genotype-3 occurred at a very high frequency (61.7%) compared to the other genotypes.

### 3.2. Serum miR-192 and miR-29a Profile

**Figure 1:**
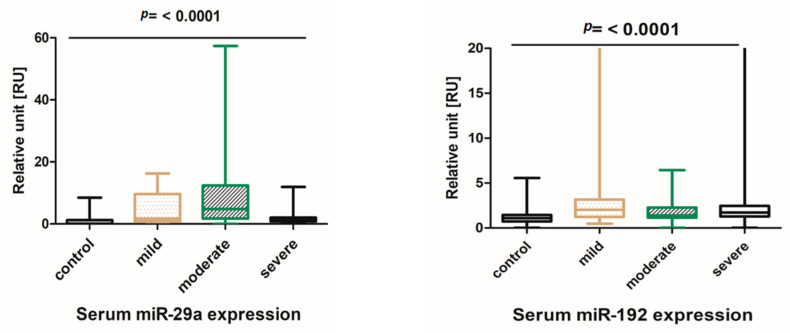
Serum expression profiles of microRNA-192 and 29a in HCV patients. Serum miRNA-192 and miR-29a in patients with mild, moderate or severe liver injury. The Whisker box plots show a significant difference between the levels of miR-192 and miR-29a in the groups of patients with different grades of liver damage.

In this study, the expression profiles of the circulating microRNAs miR-192 and miR-29a were quantified by quantitative PCR. The levels of patients with mild, moderate, and severe HCV hepatitis were compared to those in the control group of healthy blood donors. The parametric and non-parametric analyses of serum miR-192 and 29a levels both revealed that miRNAs were significantly increased compared to the controls, as shown in Figure 1. Importantly, the serum miR-29a and miR-192 levels of each patient group developing mild and moderate stages of liver injury were significantly elevated (Supplementary Figures S1 and S2).

**Figure 2:**
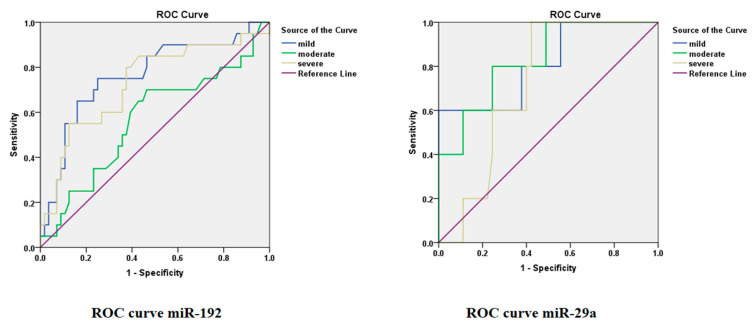
The ROC curve under the AUC for miR-192 and miR-29a in the HCV group patients.

The receiver-operating characteristic (ROC) curve under the area of (AUC) curve analysis was performed to measure the potential of individual microRNA to discriminate the HCV group with progressive liver injury. The AUC values of miR-192 and 29a showed a significant difference between the HCV-infected cohorts with moderate, mild, and severe liver injury versus the control group (Supplementary Figures S3 and S4, and Supplementary Tables S2 and S3). Figure 2 shows the ROC curve of the HCV-infected cohorts compared to the control, demonstrating that miR-192 and 29a have great potential to estimate the progression of HCV-mediated hepatic disease. The miR-192 ROC curve demonstrated that the miR-192 levels are a more sensitive indicator for mild liver injury than for moderate and severe liver damage. Similarly, the miR-29a ROC curve of the moderate group showed a particularly high sensitivity in HCV patients with mild liver injury. Overall, the analysis revealed that microRNA-29a has a more significant accuracy in diagnosis as compared to microRNA-192 in HCV patients. Therefore, the sensitivity rate of the ROC under the AUC analysis of miR-192 and 29a was considered to be significant, and their diagnostic and precision strength was substantial in HCV patients (Figure 2). According to the Youden index for microRNA 192 HCV patients, the optimal cut-off value was 1.730, the corresponding specificity of 0.65 and sensitivity of 0.8393 were reported in mild HCV patients. For moderate patients, the optimal cutoff value was 1.110, corresponding specificity 0.51 and sensitivity was 0.7778. The optimal cutoff value was 1.395, and the corresponding specificity of 0.67 and sensitivity of 0.7321 were reported in severe liver patients. Similarly, in the microRNA-29a HCV patients, the Youden index of the optimal cutoff value was 0.6250, and the corresponding specificity (0.67) and sensitivity (1.00) were noted in mild patients. For the moderate HCV group, the optimal cutoff value was 1.660, the corresponding specificity was 0.8372 and the sensitivity was 0.7907. The optimal cutoff value was 0.2400, with the corresponding specificity of 0.58 and sensitivity of 1.00, in severe HCV patients.

### 3.3. Correlation of miR-192 and 29a with ALT

Next, we compared the miR-29a and the miR-192 levels with the ALT values of patients in the moderate, mild and severe groups. The Pearson and Spearman correlation were both not significant between the miR-29a or miR-192 levels in the control or HCV-infected groups or with ALT values.

### 3.4. Expression of miR-192 and miR-29a in HCV Genotypes 3 and Non-Genotypes 3

**Figure 3:**
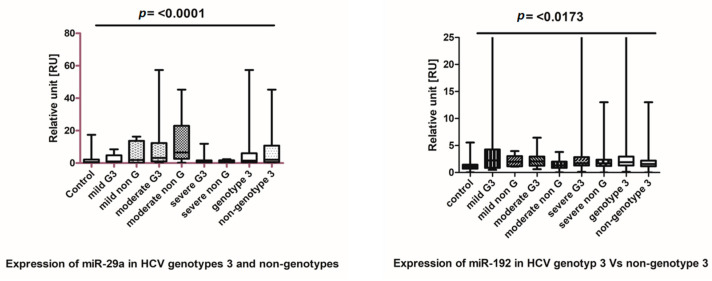
MicroRNA-29a and miR-192 profiles in the HCV genotype 3 and non-genotype 3. The Student test, comparing miR-29 levels of patients with mild, moderate and severe disease, either HCV genotype-3- or non-genotype-—infected, with the control group, showed a highly significant increase (*p* > 0.001). The significant increase in the miR-192 levels was 0.0173, and in the patient groups with mild, moderate and severe infection, we observed *p* values of 0.004, 0.015, 0.0002 and 0.013, respectively, by Student *t*-test.

Since, in the studied cohort, we observed a high frequency of patients with genotype-3, we next investigated if there was a difference between miR-29a and miR-192 levels in patients with HCV genotype-3 infections versus patients with non-genotype-3 infections. Micro-RNA-29a levels in mild, moderate and severe patients were compared with the control cohort using Student’s *t*-test. Compared to the control group, the miR-29a levels were significantly elevated in both patient groups with genotype-3 and non-genotype-3 HCV infections (Figure 3). The Bartlett’s test in the one-way ANOVA confirmed the genotype-independent increase in miR-29a, showing an equal variance and a strong correlation between miR-29a levels in patients infected with HCV genotype-3 and non-genotype-3 (Figure 3).

Moreover, miR-192 was also significantly increased in patients with genotype-3 and non-genotype-3 versus healthy controls. In Figure 3, Bartlett’s test showed that miR-192 was significantly upregulated (*p* = 0.0173) in comparison to healthy controls.

### 3.5. ROC Analysis in miR-192 and miR-29a in the HCV Genotypes 3 and Non-Genotypes 3

**Figure 4:**
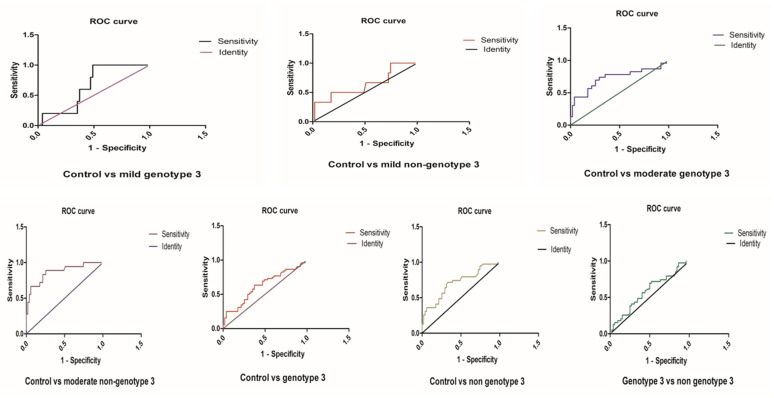
The ROC curve analysis of miR-29a in different groups of HCV patients.

The ROC analysis showed no significant difference between the comparisons of miR-29a levels in the mild genotype-3 and mild non-genotype-3 groups compared with the control group. However, in comparison to the control group, the ROC curves showed a significant difference between the HCV genotype-3 and non-genotype-3 groups with moderate fibrosis (*p* = 0.0018 and *p* < 0.0001, respectively). ROC curves for the miR-29a levels of patients with genotype-3 or non-genotype-3 in comparison to healthy controls showed significance (*p* = 0.030 or *p* = 0.0007, respectively) (Figure 4 and Table 1). No significant difference was observed in the miR-29a ROC curves for genotype-3 and non-genotype-3.

In agreement with the data of miR-29a expression in HCV patients with genotype-3 versus non-genotype-3 infections, ROC analysis indicated that miR-29a might serve as an indicator for the HCV-associated progression of fibrosis independently of the HCV genotypes (Figure 4 and Table 1).

**Figure 5:**
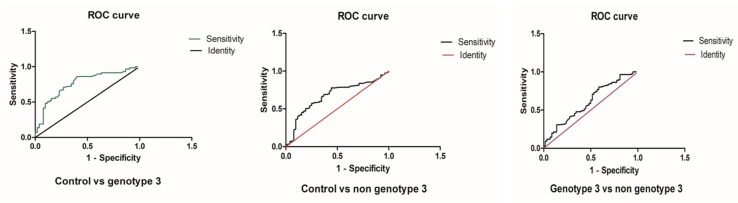
The ROC curve analysis of miR-192 in genotype 3, non-genotype 3 of HCV patients compared with the control group.

**Figure 6:**
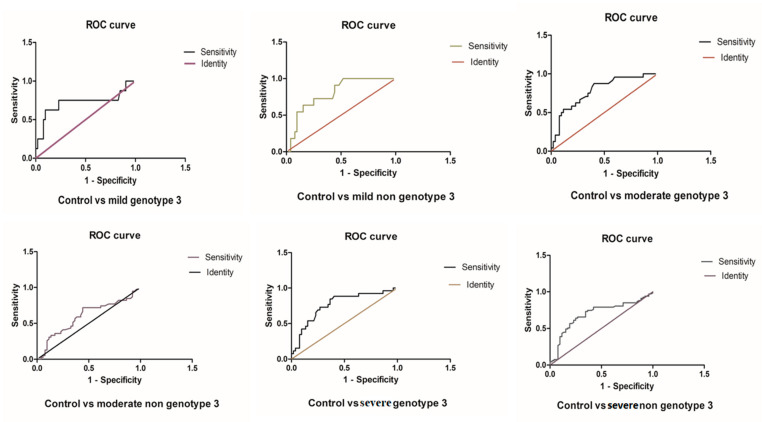
The ROC curve analysis of miR-192 with different genotypes of HCV patients.

The ROC analysis under the AUC demonstrates that miR-192 is significant in genotype-3 (*p* = 0.0001) and non-genotype-3 (*p* = 0.0003) in the control group. No significant expression was observed in the miR-192 and miR-29a ROC curve between HCV genotype-3 and non-genotype-3, as shown in Figure 5 and Table 2. Similarly, no significant difference was observed in the miR-192 ROC curve of moderate non-genotype-3 with the control group. Still, the other groups of miR-192 disclosed significant upregulation with healthy controls (Figure 6 and Supplementary Table S2). Interestingly, the ROC data showed no difference in the potential of miR-192 to indicate infections with HCV genotype-3 versus non-genotype-3 (Figure 5 and Table 2).

## 4. Discussion

The present study focused on circulating miR-192 and 29a profiles in patients with hepatitis C disease and the comparative analysis at different stages of liver progression/injury. Various studies revealed that miR-192-5p and the miR-29 family are involved in human diseases, such as cancers, liver, kidney, nervous, heart and breast. Significantly, miR-192 and 29a levels are abundant in the urine and serum, and the exosomal stages in the circulation can help to diagnose and predict different diseases, such as hepatic disease, mostly those involving hepatitis infections [23,32]. MicroRNA-29a is known to act as antifibrotic miRNA, and its hepatic loss and secretion during liver disease are shown to be associated with liver fibrosis [23,24,26]. Extracellular miR-192 is induced after metabolic liver disease and HCV-mediated hepatitis and participates in inflammatory processes [32]. Many causative agents damage the liver, but microRNA-192-5p plays a role as a biomarker for diagnosis during hepatic injury. Thus, Roy et al. studied miR-192-5p in the serum of mice and human liver injury. The authors conclude that this miRNA was significantly upregulated in hepatic disease, but, in contrast, downregulated in the liver tissue [33].

In our study, samples of 222 patients with chronic hepatitis C infection were used to compare miR-192 and miR-29a serum levels with those of healthy controls. Both miRNAs, mir-29a and miR-192, were elevated during the liver disease progression of HCV patients. This was in agreement with other reports by Ezaz et al. [29] and Liu et al. [28], which have shown that miR-192-5p and the family 29a are significantly decreased in liver tissue but elevated in serum samples of patients with liver fibrosis [28,29]. The ROC analysis and area under the curve (AUC) were used to assess the diagnostic performance and accuracy of the miR-192 and 29a. Here, we noted that these microRNAs show a significant ROC performance and AUC curve in HCV patients. In the comparative analysis, the mild and moderate groups significantly outperformed the miR-192 and miR-29a, respectively. Overall, comparing these microRNAs showed a significant sensitivity rate in diagnosing hepatic disease. The ROC and AUC revealed that these microRNAs might be used as a biomarker to indicate the prognosis and severity of the liver injury. Similarly, the study of Motawi [34] analyzing the ROC and AUC in chronic HCV patients was closely related to our results. Several approaches and techniques have been used to identify and quantify microRNAs, resulting in discrepancies between reported results. One study found that miR-192 was elevated in HCV-infected patients with cirrhosis relative to those without cirrhosis and that it may boost HCV replication by targeting host genes involved in lipid metabolism. Another study demonstrated that miR-29a was downregulated in HCV-infected cirrhotic individuals and could impede HCV replication by targeting the 3′ UTR of the viral genome. A different study demonstrated that miR-29a could promote HCV replication by targeting host genes involved in lipid metabolism [35,36]. In light of these contradictory findings, the role of miRNA 192 and miRNA 29a in HCV replication remains unknown, and further research is required to identify their diagnostic value as biomarkers for HCV infection.

Most investigations indicate that miR-29a may function as an antiviral host factor by inhibiting HCV replication, and may have therapeutic promise for treating HCV infection. In addition, the overexpression of miR-192 and miR-29a in HCV patients may be attributable to their role in regulating fibrosis and inflammation, which are prominent characteristics of chronic liver disease [35,36]. MiR-192 and miR-29a may be possible non-invasive biomarkers for diagnosing fibrosis progression after chronic liver disease caused by HCV infection, as our investigation supports [36,37]. The causes of the variations in miR-192 and miR-29a levels between liver tissue and serum samples are unknown. It has been argued that sample preparation and analysis procedure variations may contribute to these disparities. Consequently, more studies are needed to establish the causes of these inconsistencies and to develop standardized methodologies for assessing miRNA concentrations in various sample types.

Regardless of the HCV genotype, our study provides more data supporting the potential of miR-192 and miR-29a as biomarkers for hepatic disease. These biomarkers may be useful for identifying the advancement of fibrosis in patients with chronic liver disease due to HCV infection [37,38]. Further research is necessary to assess the therapeutic efficacy of these biomarkers and to develop standardized methods for detecting miRNA levels in diverse sample types. Recent research has studied the diagnostic potential of miR-192 and miR-29a as biomarkers for HCV patients, although the findings are limited. One such limitation is the pilot study’s relatively small sample size, which necessitates higher sample sizes to confirm the diagnostic usefulness of these miRNAs.

Various challenges must be overcome to fully comprehend the diagnostic biomarker potential of microRNAs [8,9]. The lack of standardization in miRNA isolation and quantification, inconsistencies in sample preparation, the presence of contaminants and inhibitors, a limited understanding of miRNA biology, and the absence of large-scale validation studies to determine the reliability and robustness of miRNA-based diagnostic tests are among these impediments.

Future studies could emphasize the diagnostic and prognostic capabilities of miR-192 and miR-29a in expanded and more diverse patient populations. Investigating miRNA expression in various HCV genotypes and disease phases could also shed light on the potential use of miRNAs as biomarkers for hepatic disease. In addition, understanding the mechanisms underlying the overexpression of miR-192 and miR-29a throughout chronic HCV infection could reveal prospective therapeutic targets for treating HCV-related liver disorders. By conquering these challenges and pursuing future research routes, we can improve the translation of miRNA research into clinical practice, resulting in patient outcomes.

Overall, our study demonstrates that themiR-29a and miR-192 s are significantly upregulated in patients with HCV genotype-3 and non-genotypes-3 in a similar pattern. Thus, both miRNAs could be an indicator of hepatic disease progression independently of the HCV genotype.

## 5. Conclusions

The presented study reveals that serum microRNA-192 and miR-29a are significantly increased in patients with chronic hepatic disease infected by HCV genotype 3 and non-genotype 3. Notably, these miRNAs appear to be a sensitive predictor of liver disease and may be better-suited than other regular biochemical assays to monitoring HCV-related liver injury.

## Figures and Tables

**Table 1:** MicroRNA-29a performance in HCV patients.

S. No.	Area under the ROC Curve	95% Confidence Interval	Std. Error	*p* Value
Control–Mild genotype 3	0.6549	0.4763 to 0.8335	0.09108	0.2564
Control–Mild non-genotype 3	0.6356	0.3783 to 0.8929	0.1312	0.2806
Control–Moderate genotype 3	0.7276	0.5866 to 0.8686	0.07193	<0.001829
Control–Moderate non-genotype 3	0.8687	0.7674 to 0.9700	0.05168	<0.0001
Control–Severe genotype 3	0.5176	0.3789 to 0.6562	0.07073	0.8071
Control–Severe non-genotype 3	0.5438	0.3880 to 0.6996	0.07949	0.6083
Control vs. Genotype 3	0.6237	0.5152 to 0.7321	0.05532	<0.03055
Control vs. Non-genotype 3	0.7079	0.5987 to 0.8171	0.05569	<0.0007660
Genotype 3 vs. Non-genotype 3	0.5816	0.4625 to 0.7007	0.06076	0.1845

**Table 2:** MicroRNA-192 performance in HCV patients.

S. No.	Area under the ROC Curve	95% Confidence Interval	Std. Error	*p* Value
Control–Mild genotype 3	0.7200	0.4736 to 0.9663	0.1256	<0.04667
Control–Mild non-genotype 3	0.7981	0.6735 to 0.9227	0.06357	<0.002033
Control–Moderate genotype 3	0.7800	0.6703 to 0.8898	0.05596	<0.0001
Control–Moderate non-genotype 3	0.5971	0.4759 to 0.7184	0.06186	0.1142
Control–Severe genotype 3	0.7618	0.6461 to 0.8776	0.05905	<0.0001772
Control–Severe non-genotype 3	0.6945	0.5969 to 0.7920	0.04976	0.0002866
Control vs. Genotype 3	0.7636	0.6728 to 0.8544	0.04631	<0.0001
Control vs. Non-genotype 3	0.6718	0.5852 to 0.7583	0.04416	0.0003750
Genotype 3 vs. Non-genotype 3	0.6102	0.5206 to 0.6997	0.04568	0.01785

## Data Availability

All data generated or analyzed during this study are included in the manuscript and its supplementary files.

## Supplementary Materials

The following supporting information can be downloaded at: https://www.mdpi.com/article/10.3390/genes14051056/s1, Table S1: (A): The demographic and clinical features of the enrolled patients; (B): LFTs, Genotypes, Risk factors of chronic HCV Patients; (C): List of healthy controls (n = 52), used in our study; Table S2: MicroRNA-192 performance in HCV patients; Table S3: MicroRNA-29a diagnostic performance in HCV patients; Figure S1. microRNA-192; Figure S2. microRNA-29a; Figure S3. ROC-192; Figure S4. ROC-29a.
