# Supplementary material for: Circulating miRNA-192 and miR-29a as Disease Progression Biomarkers in Hepatitis C Patients with a Prevalence of HCV Genotype 3
Source: Genes (Basel). 2023 May 8;14(5):1056. doi: 10.3390/genes14051056 (PMC10217963; doi:10.3390/genes14051056)
Supplement: Supplementary file 1 [file genes-14-01056-s001.zip › genes-2238683-supplementary.pdf]

Supplementary Table S1A. The demographic and clinical features of the enrolled patients (n=222).

No. of Variables	No. of Values
**Gender**	
Gender	Frequency
Male	61%
Female	39%
**Age, years**	
Age	Frequency
<30	15
31-50	112
51-70	90
>70	05
Average	49.03
Range-years ± SD	19-91 ± 12.65
Healthy control (52)	
Gender	Frequency
Male	28 (54%)
Female	24 (46%)
Average age	40
Mean±SD- years	40±12.20
**Age, years**	
<30	15(29%)
31-50	26(50%)
51-70	10(19%)
>70	1(2%)
**HCV infected patients**	
Mild (n)<br>mean± SD years<br>mode	37<br>50.56± 13.19<br>50
Moderate (n)<br>mean± SD years<br>Mode	87<br>47.18± 12.25<br>55
Severe (n)<br>mean± (SD years<br>mode	98<br>48.0± 11.94<br>50

Child Turcotte Pugh Score		
Child score A (5-6)	17%	
Child score A (7-9)	39%	
Child score A (10-15)	44%	
**Ascites**		
Male	High	38.2%
	Moderate	16.9%
	Less	4.5%
Female	High	20.6%
	Moderate	13.5%
	Less	6.4%
**Blood pressure**		
Male	Normal	18%
	High	15.4%
	Low	26.2%
Female	Normal	15%
	High	10.9%
	Low	14.6%
**Size of spleen**		
Male	Normal	21%
	Enlarged	38.6%
Female	Normal	15%
	Enlarged	25.5%
**GIT**		
Male	Normal	4%
	Tenderness	21.3%
	Non-tenderness	37.8%
Female	Normal	7%
	Tenderness	12%
	Non-tenderness	27.7%
**Viral load (Iu/ml)**		
<10,00000	49.4%	
10,00000 -50,00000	26.21%	
>50,00000	24.34%	

Abbreviation: GIT; gastro-intestinal tract.

Supplementary Table S1B. LFTs, Genotypes, Risk factors of chronic HCV Patients.

**ALT/AST**		
Average –Median	121.46 – 121	
Mode	139	
Range ± Standard deviation	64-186 ± 29.23	
**AFP**		
<40 ng/mL	33%	
41-150 ng/mL	47%	
>151 ng/mL	30%	
Mean	43.09	
Range ± Standard deviation	16-301±28.08	
**Albumin**		
Male	Normal (3-5 g/dL)	15.7%
	Abnormal (<3-5 g/dL)	24.7%
Female	Normal (3-5 g/dL)	19.1%
	Abnormal (<3-5 g/dL)	40.4%
**Risk factors**		
Dentist	33.08%	
Unknown source	28.95%	
Surgery	10.86%	
HCV in family	23.97%	
Blood Transfusion	4.11%	
**Genotypes**		
Genotype 1	11(5)	
Genotype 2	23(10.4)	
Genotype 3	137(61.7)	
Mixed	9(4.1)	
Untyped	42(18.9)	
**Viral load (Iu/ml)**		
<10,00000	49.4%	
10,00000 -50,00000	26.21%	
>50,00000	24.34%	

Abbreviation: ALT; alanine aminotransferase. **AST**; aspartate aminotransferase. **AFP;** alpha fetoprotein. **ALP**; alkaline phosphatase

Supplementary Table S1C. List of healthy control (n=52) used in the HCV study.

S.No	Age	Gender	Area
1	50	F	Peshawar
2	48	M	Charsada
3	30	F	Peshawar
4	33	M	Peshawar
5	43	M	Peshawar
6	33	F	Peshawar
7	35	F	Mardan
8	60	M	Peshawar
9	40	F	Peshawar
10	55	M	Peshawar
11	65	M	Peshawar
12	48	M	Charsada
13	30	F	Peshawar
14	29	F	Mardan
15	50	M	Peshawar
16	50	M	Peshawar
17	40	F	Peshawar
18	35	F	Mardan
19	61	M	Peshawar
20	28	F	Peshawar
21	25	M	Peshawar
22	39	F	Peshawar
23	44	M	Peshawar
24	25	F	Peshawar
25	50	M	Peshawar
26	45	M	Peshawar
27	30	M	Charsada
28	79	F	Peshawar
29	40	M	Mardan
30	49	M	Peshawar
31	30	M	Peshawar
32	62	M	Peshawar
33	30	F	Peshawar
34	45	F	Malakand
35	45	M	Peshawar
36	36	M	Peshawar
37	40	F	Peshawar
38	44	M	Mardan
39	52	F	Mardan

40	30	F	Swabi
41	40	M	Peshawar
42	40	F	Mardan
43	52	M	Charsada
44	50	F	Charsada
45	23	F	Peshawar
46	22	M	Mardan
47	40	F	Peshawar
48	39	M	Kohat
49	23	F	Mardan
50	20	F	Peshawar
51	45	M	Kohat
52	27	M	Peshawar

Supplementary Table S2. MicroRNA- 192 performance in HCV patient.

Parameters	Area under the ROC curve	95% confidence interval	Std. Error	P value
Control- Mild	0.7625	0.6331 to 0.8919	0.06601	< 0.0005
Control- Moderate	0.6590	0.5601 to 0.7579	0.05046	< 0.0028
Control- Severe	0.7076	0.6209 to 0.7942	0.04420	< 0.0001
Mild- Moderate	0.6123	0.4705 to 0.7541	0.07231	0.1320
Mild- Severe	0.7625	0.6331 to 0.8919	0.06601	< 0.0005
Moderate- Severe	0.5555	0.4624 to 0.6485	0.04747	0.2405

Supplementary Table S3. MicroRNA- 29a diagnostic performance in HCV patient.

Parameters	Area under the ROC curve	95% confidence interval	Std. Error	P value
Control- Mild	0.8349	0.7181 to 0.9517	0.05958	< 0.0010
Control- Moderate	0.8588	0.7791 to 0.9386	0.04067	< 0.0001
Control- Severe	0.7862	0.6834 to 0.8889	0.05241	< 0.0001
Mild- Moderate	0.6081	0.4264 to 0.7899	0.09271	0.2905
Mild- Severe	0.8349	0.7181 to 0.9517	0.05958	0.05958
Moderate- Severe	0.7462	0.6308 to 0.8616	0.05886	0.00010

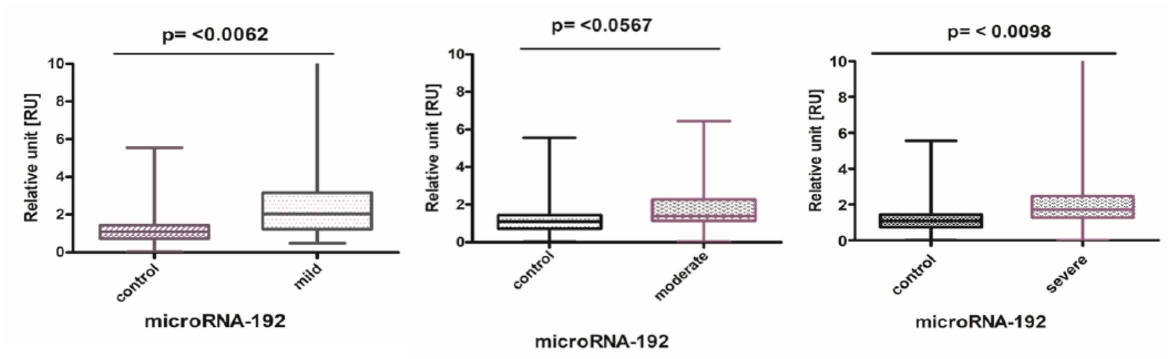

Figure S1. microRNA-192.

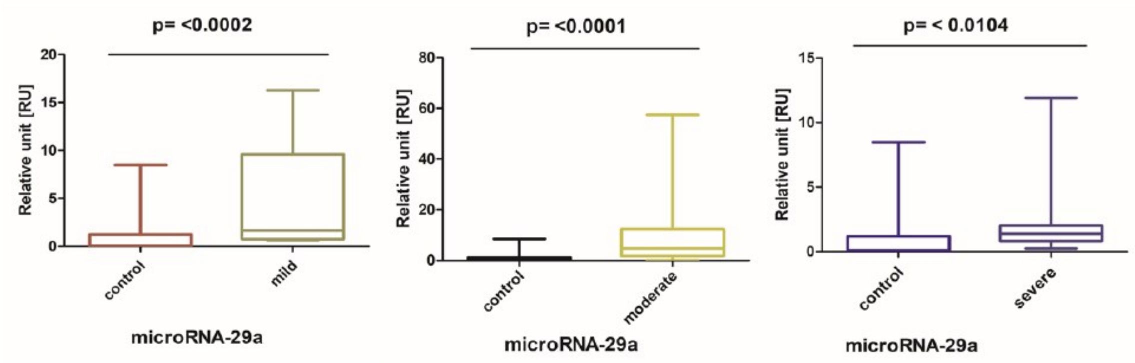

Figure S2. microRNA-29a.

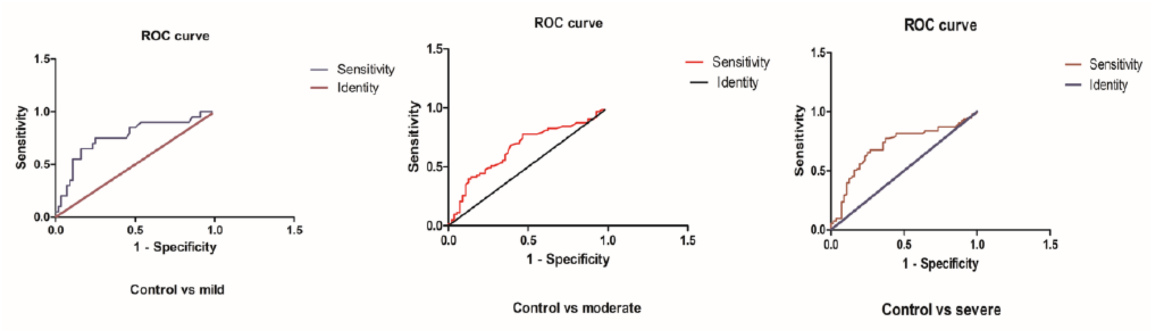

ROC-192

Figure S3. ROC-192.

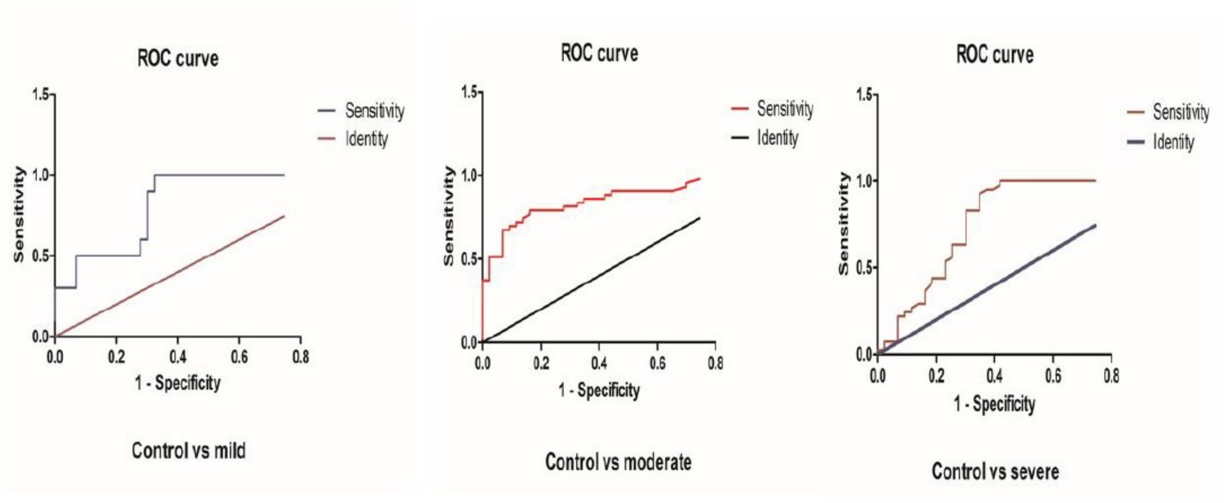

Figure S4. ROC-29a
